# Factors influencing the uptake of a mass media intervention to improve child feeding in Bangladesh

**DOI:** 10.1111/mcn.12603

**Published:** 2018-04-11

**Authors:** Sunny S. Kim, Terry Roopnaraine, Phuong H. Nguyen, Kuntal K. Saha, Mahbubul I. Bhuiyan, Purnima Menon

**Affiliations:** ^1^ Poverty, Health, and Nutrition Division International Food Policy Research Institute Washington District of Columbia USA; ^2^ Oxford Policy Management Oxford UK; ^3^ Department of Nutrition for Health and Development World Health Organization Geneva Switzerland; ^4^ Poverty, Health, and Nutrition Division International Food Policy Research Institute Dhaka Bangladesh; ^5^ Poverty, Health, and Nutrition Division International Food Policy Research Institute New Delhi India

**Keywords:** Bangladesh, behaviour change communication, infant and young child feeding, mass media, television

## Abstract

Mass media are increasingly used to deliver health messages to promote social and behaviour change, but there has been little evidence of mass media use for improving a set of child feeding practices, other than campaigns to promote breastfeeding. This study aimed to examine the factors influencing the uptake of infant and young child feeding messages promoted in TV spots that were launched and aired nationwide in Bangladesh. We conducted a mixed‐methods study, using household surveys (*n* = 2,000) and semistructured interviews (*n* = 251) with mothers of children 0–23.9 months and other household members. Factors associated with TV spot viewing and comprehension were analysed using multivariable logistic regression models, and interview transcripts were analysed by systematic coding and iterative summaries. Exposure ranged from 36% to 62% across 6 TV spots, with comprehension ranging from 33% to 96% among those who viewed the spots. Factors associated with comprehension of TV spot messages included younger maternal age and receipt of home visits by frontline health workers. Three direct narrative spots showed correct message recall and strong believability, identification, and feasibility of practicing the recommended behaviours. Two spots that used a metaphorical and indirect narrative style were not well understood by respondents. Understanding the differences in the uptake factors may help to explain variability of impacts and ways to improve the design and implementation of mass media strategies.

**Key messages**

- Despite higher exposure and prior knowledge of the TV spot messages in the intensive intervention areas compared with nonintensive areas, there was little difference in comprehension.
- Direct narrative spots showed correct message recall and strong believability, identification, and feasibility of practicing the recommended behaviours; whereas the spots that used a metaphorical and indirect narrative style were not well understood.
- For behaviours highly embedded in sociocultural contexts such as child feeding, well‐designed mass media interventions that reach families and the broader community play an important role in combination with other strategies, and more evidence is needed to refine these interventions for social and behaviour change.

## 1 INTRODUCTION

Despite rapid improvements in maternal and infant mortality and other health and development indicators in Bangladesh (Chowdhury et al., 2013), close to one third of children less than 5 years of age remain undernourished (NIPORT, Mitra and Associates, & ICF International, 2016). Appropriate infant and young child feeding (IYCF) practices are critical for child growth and development (Bhutta et al., 2013; Dewey & Adu‐afarwuah, 2008; Haroon, Das, Salam, Imdad, & Bhutta, 2013; Lassi, Das, Zahid, Imdad, & Bhutta, 2013). However, only about half of Bangladeshi children less than 6 months of age are exclusively breastfed. Nonbreast milk liquids and foods are introduced either too early or too late, and the quality of complementary feeding is poor, with barely one‐quarter of children aged 6–23 months of age consuming adequately diverse foods (NIPORT et al., 2016).

Various interventions are proven effective to support better IYCF practices; interpersonal counselling and group meetings to provide education and/or support for mothers have been shown to increase exclusive breastfeeding practices (Haider, Kabir, Huttly, & Ashworth, 2002; Haroon et al., 2013; Imdad, Yakoob, & Bhutta, 2011a; Sinha et al., 2015). Nutrition education about complementary feeding with or without provision of food supplements have demonstrated impacts on child growth (Caulfield, Huffman, & Piwoz, 1999; Dewey & Adu‐afarwuah, 2008; Imdad, Yakoob, & Bhutta, 2011b; Lassi et al., 2013), including in Bangladesh (Roy et al., 2005; Saha et al., 2008).

Mass media (TV, radio, billboard, newspaper, and other print or electronic materials) are increasingly used to deliver health messages to promote social and behaviour change, as they have the potential to modify the knowledge and attitudes of large proportions of the population simultaneously (Jepson, Harris, Platt, & Tannahill, 2010; Snyder, 2007; Wakefield, Loken, & Hornik, 2010). Mass media interventions have shown to be effective in reducing smoking behaviour (Bala, Strzeszynski, & Cahill, 2008; Brinn, Carson, Esterman, Chang, & Smith, 2010), increasing consumption of healthy foods (Matson‐Koffman, Brownstein, Neiner, & Greaney, 2005; Pomerleau, Lock, Knai, & McKee, 2005), and increasing physical activity (Conn, Hafdahl, & Mehr, 2011; Kahn et al., 2010), particularly in the context of developed countries. There is also growing evidence of mass media use in developing countries for the promotion of family planning and reproductive health (Piotrow et al., 1997) and promotion of child survival particularly through vaccination (Hornik et al., 2002; Hutchinson, Lance, Guilkey, Shahjahan, & Haque, 2006; Pegurri, Fox‐Rushby, & Damian, 2005). Reviews of mass media interventions have concluded that well‐developed messages for target audiences had a higher success rate; intensity and duration influence effectiveness; and implementation with combined strategies contribute to successful campaigns; but results vary significantly depending on length of follow‐up, methodology, and context of behaviours (e.g., social norms, habits/addiction, and product marketing; Jepson et al., 2010; Snyder, 2007; Wakefield et al., 2010). Despite these various studies, there has been little evidence of mass media use for improving a set of IYCF practices, other than campaigns to promote breastfeeding (Nguyen et al., 2016; Thompson & Harutyunyan, 2009; Wilmoth & Elder, 1995).

This paper aims to examine the factors influencing the uptake of IYCF messages promoted in TV spots that were launched and aired nationwide in Bangladesh between 2011 and 2013. We assessed exposure to the TV spots, comprehension of key messages, and factors associated with uptake of messages, including TV viewing barriers and facilitators, believability, identification, and feasibility of behaviours recommended in each TV spot. In a mixed‐methods study design, we used quantitative household survey data and in‐depth qualitative interviews to explore this set of issues comprehensively in the context of a large‐scale social and behaviour change program.

### 1.1 Alive & Thrive interventions in Bangladesh

Alive & Thrive (A&T) is a multiyear initiative aimed at reducing undernutrition and death caused by suboptimal IYCF practices. In Bangladesh, A&T used a combination of behaviour change communication strategies: interpersonal communication, mass media, and social mobilization to promote adequate IYCF practices and create an enabling environment for mothers and caregivers to adopt the recommended practices (Sanghvi et al., 2016).

BRAC, a large non‐governmental organization, delivered intensified IYCF counselling and community mobilization activities through its existing essential health care program. BRAC's extensive network of frontline health workers (FHW) conducted multiple visits to households with pregnant women and children less than 2 years of age to provide age‐targeted IYCF counselling. Community mobilization included awareness‐building activities such as meetings and forums with husbands, religious leaders, health committee members, and other community leaders; and community theatre shows focused on IYCF topics. In February 2011, A&T also launched a national television and radio campaign. The TV campaign included six high‐quality spots with messages on breastfeeding (two spots) and complementary feeding issues (four spots) that were identified as gaps in current practices, based on formative research (Sanghvi et al., 2016; Sanghvi, Jimerson, Hajeebhoy, & Zewale, 2013). The description of the six spots are presented in Table 1. Strategies for “media dark” areas without adequate TV coverage, such as community video showings, were implemented in certain hard‐to‐reach and low‐electricity communities in the intervention areas. The TV spots were broadcast through national channels until the end of 2013.

**Table 1:** Description of the six TV spots^a^

TV spot	Description
Breastfeeding‐focused spots:	
1. Early initiation of breastfeeding (BF)	*Direct messaging:* Having just given birth, a young mother immediately asks for her baby to be brought to her so that she can begin BF right away, per the doctor's instructions and against the advice of an older woman who attempts to administer honey as a prelacteal. This spot emphasizes the importance of initiating BF within the first hour or life.
2. Perception of insufficient milk	*Direct messaging*: A well‐meaning father returns home with a tin of formula milk that he has purchased for his 3‐month‐old baby. This is rejected by the mother, who explains that the baby should receive only breast milk at this stage. Here, an older woman is cast as a wise voice, supporting the mother's claim that breast milk is best for the child. This spot addresses the misperception that feeding a child only breast milk is not enough.
Complementary feeding‐focused spots:	
3. Father's involvement	*Indirect messaging:* A son responds quickly in the face of a dangerous situation (a house fire), saving his father's life. The father has a flashback of the son being fed well from infancy and of helping his wife to care for his son. This spot depicts the role fathers can play in the growth and development of their child in the first 2 years.
4. Animal food	*Direct messaging*: A mother feeds her child fish from a pot, then her neighbour arrives and questions whether fish should be given to a child so young. The mother responds that she was instructed to feed the child in this way by her doctor. This spot promotes incorporating animal source foods in a baby's diet.
5. Feeding quantity	*Indirect messaging*: A primary schoolgirl named Tumpa is shown receiving prizes for sporting and academic achievements. Asked about the secret of her success by another mother, Tumpa's proud mother explains that she made sure that Tumpa was eating the right kinds of foods in the right quantity when she was younger. This spot promotes giving the proper amount of food to children at various ages to ensure optimal growth and development.
6. Poor appetite	*Direct‐messaging*: This spot addresses the question of what to do when the child has a poor appetite. Parents are discouraged from forcing a reluctant child to eat, and they are steered away from feeding junk foods, store‐bought snacks, and juices. They are encouraged by a white‐coated doctor figure to feed children a variety of foods, on demand.

a Adapted from descriptions and video clips of the TV spots in http://www.aliveanthrive.org.

## 2 METHODS

### 2.1 Household survey

This study used data from a cross‐sectional household survey conducted in April–May 2013, as part of the A&T process evaluation (Rawat et al., 2013). The survey sample was based on the sampling method for the cluster‐randomized impact evaluation design used to evaluate the A&T interventions (Menon, Rawat, & Ruel, 2013). Twenty rural *upazilas* (subdistricts) with BRAC's essential health care program were randomly selected to receive A&T's intensified community interventions plus mass media (10 upazilas with *intensive* interventions) or the standard of care with mass media only (10 upazilas with *nonintensive* interventions). The household survey consisted of interviews with mothers of children 0–23.9 months of age (*n* = 2,000) about their frequency of TV watching, exposure to interventions, and other factors. An aided recall method was applied using screen shots from the six TV spots; mothers were shown the images, then asked about their exposure to each TV spot (whether they ever viewed the spot), prior knowledge of the TV spot messages (whether they had heard the spot messages before), and comprehension of the TV spots (identification of main messages per spot, asked by “what is the spot asking you to do?”).

### 2.2 Qualitative research methods

Focused ethnographic research (Cove & Pelto, 1993; Pelto, Armar‐Klemesu, Siekmann, & Schofield, 2013) was used to elucidate how the A&T TV spots were received and understood by caregivers, including issues of comprehension, believability (whether message was delivered by credible authority figures and in agreement with information from other sources), identification (whether scenarios were of common or relevant circumstances and characters had strong relatability or acceptance), feasibility of the promoted behaviours (whether caregiver had capability to do or accomplish), and, when manifested, prior knowledge (Salmon & Atkin, 2003). These principles for public communication campaigns have been suggested for purposely informing and influencing behaviours and were adapted for this study. Aided recall was used by showing respondents each of the six TV spots once on a laptop, followed by a series of open‐ended questions about the content, messages, and related opinions, particularly about each of the principles defined above.

The qualitative research was conducted in three upazilas drawn from the impact evaluation sample: two upazilas with intensive interventions (Madhabpur and Sonaimuri) and one with nonintensive interventions (Chirirbandar). Based on discussions with the A&T country program staff and BRAC, these upazilas were selected purposively to meet several criteria, including geographic diversity and known conservative areas (where program uptake could face more challenges and hence having data would be of good use). Three villages were selected per upazila, for a total of nine villages. Households within these villages were listed using participatory rural appraisal techniques for social mapping (Chambers, 1994). Then, 10 households (*n* = 90) were selected in each village to ensure that the two age groups for IYCF interventions were covered (i.e., 30 households with children 0–5.9 months and 60 households with children 6–23.9 months). Within each sampled household, the mother and, where possible, the father and grandmother or elderly woman participated in a semistructured interview (*n* = 251), thus enabling us to capture and document insights related to the intervention components through different perspectives of household members. Data collection was conducted between January and September 2012.

### 2.3 Data analysis

Results of exposure and recall of correct messages for each TV spot between program groups were compared using chi‐square tests. Factors associated with TV spot viewing and comprehension were assessed using multivariable logistic regression models in the overall sample, controlling for clustering at the upazila level. Quantitative data analysis was done using Stata 14. We do not analyse for the impact of interventions on behavioural outcomes, which are presented in a separate paper (Menon et al., 2016).

For qualitative data analysis, interview transcripts were transcribed in verbatim from audio recordings and translated from Bangla to English. A detailed a priori descriptive code list was drawn up based on the key research questions and concepts of interest, then further refined based on emergent themes. Key themes included TV viewing barriers and facilitators, recalled elements, believability, identification, and feasibility of recommended behaviour of each TV spot. Content analysis and systematic coding were undertaken using NVivo 10 software. Outputs of coded results using queries, or code‐based search commands, were generated to elaborate iterative summaries of findings.

Informed consent was obtained from all study participants. Ethical clearance for this research was received from the Bangladesh Medical Research Council and Institutional Review Board at International Food Policy Research Institute.

## 3 RESULTS

### 3.1 Study sample characteristics

Demographic characteristics of the sampled mothers in the household survey are presented in Table 2. Mean maternal age (about 25 years), number of children less than 5 years of age (1.3 children), education level (14% no schooling, 32% primary school, and 54% middle school or higher), occupation (79% housewives), and religion (95% Muslim) were similar between program groups. In the nonintensive areas, household socio‐economic status and receipt of home visits for IYCF counselling by any FHW were significantly lower, whereas TV ownership and frequency of TV watching was higher, as compared with intensive areas. Overall, about 40% of the households owned a TV, and nearly one‐half of the respondent mothers watched TV frequently (three or more days per week).

**Table 2:** Survey sample characteristics, by program group

	A&T‐intensive	A&T nonintensive	Total
	(*N* = 1,000)	(*N* = 1,000)	(*N* = 2,000)
Characteristic	Mean ± *SD*/percent	Mean ± *SD*/percent	Mean ± *SD*/percent
Maternal age (years)	25.21 ± 5.34	24.86 ± 5.50	25.03 ± 5.42
Number of children <5 years	1.32 ± 0.55	1.35 ± 0.57	1.34 ± 0.56
Maternal education level:			
Never attended school	13.60	14.80	14.20
Primary school (Class 1–5)	32.70	31.80	32.25
Middle school (Class 6–9)	42.60	41.70	42.15
High school or higher	11.10	11.70	11.40
Maternal occupation:			
Housewife/house work	78.10	80.30	79.20
Other	21.90	19.70	20.80
Religion			
Muslim	96.50	93.40	94.95
Hindu	3.30	6.50	4.90
Other	0.20	0.10	0.15
Household SES level:			
Very low	23.00	27.00	25.00
Low	22.80	27.20	25.00
Middle	25.00	25.00	25.00
High	29.20	20.80***	25.00
TV ownership	34.90	44.40***	39.65
Frequency of watching TV:			
None	33.10	23.40	28.25
Infrequent (<3 days/week)	22.70	21.30	22.00
Frequent (three or more days/week)	44.20	55.30***	49.75
Received any FHW visit	95.40	39.30***	67.35

* *p* < .05.

** *p* < .01.

*** *p* < .001.

The qualitative study included 90 mothers with children less than 2 years of age, 80 fathers, and 81 grandmothers. The mean age of mothers was 25 years (range 20–38), and nearly all were housewives. Mean age of fathers was 36 years (range 24–52), and most were farmers (*n* = 54). Grandmothers' mean age was 58 years (range 40–80), and most were illiterate (*n* = 62).

### 3.2 TV spot exposure, comprehension, and associated factors

Exposure to the TV spots in the intensive areas was moderate (36–62%), nearly reaching the proportion of mothers who generally watch TV, and was consistently higher by approximately 10 percentage points than in nonintensive areas for all TV spots (Table 3). Spots 1, 2, and 4 had the highest rates of exposure, whereas Spots 3, 5, and 6 had the lowest for both program groups. Among those who had seen the TV spots, prior knowledge of the spot messages was also significantly higher in intensive areas. Spots 1, 2, and 4 had high comprehension (~90%), whereas Spots 3 and 5 had the lowest rates of comprehension in both program groups. Comprehension for Spots 1 and 6 was slightly higher in the intensive areas (Table 3).

**Table 3:** Exposure, prior awareness, and comprehension of TV spots among mothers, by program group

	A&T‐intensive	A&T nonintensive	Total
	(*N* = 1,000)	(*N* = 1,000)	(*N* = 2,000)
Indicator	Percent	Percent	Percent
Ever viewed TV spot			
TV Spot 1	65.70	53.50***	59.60
TV Spot 2	62.40	50.70***	56.55
TV Spot 3	45.20	26.30***	35.75
TV Spot 4	66.90	56.70***	61.80
TV Spot 5	53.50	43.10***	48.30
TV Spot 6	56.90	42.10***	49.50
Prior awareness^a^			
TV Spot 1	64.84	32.71***	50.42
TV Spot 2	57.69	24.65***	42.88
TV Spot 3	39.60	12.17***	29.51
TV Spot 4	61.14	23.81***	44.01
TV Spot 5	43.74	10.90***	29.09
TV Spot 6	51.67	15.20***	36.16
Comprehension^a^^,^ b			
TV Spot 1	90.41	85.42***	88.17
TV Spot 2	90.54	88.95	89.83
TV Spot 3	34.29	30.80	33.01
TV Spot 4	96.71	94.89	95.87
TV Spot 5	38.69	38.28	38.51
TV Spot 6	63.62	57.01*	60.81

a Among those who viewed the TV spot.

b Identified key message of the TV spot.

* *p* < .05.

** *p* < .01.

*** *p* < .001.

Given similar results in the multivariable logistic regression models by program group (not shown), results of the factors associated with TV spot viewing and comprehension are presented for the overall sample. Factors associated with viewing the TV spots (patterns across three or more spots) included being a younger mother (less than 20 years) compared with being an older mother (more than 30 years; odds ratio [OR]: 0.6), higher education level (high school or higher; OR: 1.6–1.9), frequent TV watching (three or more days per week, OR: 4.4–19.7), and receipt of at least one home visit from a FHW (OR: 2.4–2.7; Table 4).

**Table 4:** Factors associated with viewing and comprehension of TV spots^a^^,^ b

Indicator	TV Spot 1		TV Spot 2		TV Spot 3		TV Spot 4		TV Spot 5		TV Spot 6	
	Viewing, OR	Comprehension, OR	Viewing, OR	Comprehension, OR	Viewing, OR	Comprehension, OR	Viewing, OR	Comprehension, OR	Viewing, OR	Comprehension, OR	Viewing, OR	Comprehension, OR
	(*n* = 2,000)	(*n* = 1,192)	(*n* = 2,000)	(*n* = 1,131)	(*n* = 2,000)	(*n* = 715)	(*n* = 2,000)	(*n* = 1,236)	(*n* = 2,000)	(*n* = 966)	(*n* = 2,000)	(*n* = 990)
Maternal age: (years)												
<20	Ref	Ref	Ref	Ref	Ref	Ref	Ref	Ref	Ref	Ref	Ref	Ref
20–30	0.86	0.59**	0.78*	0.78	0.97	0.89	1.07	1.11	0.97	1.00	0.85	0.91
>30	0.81	0.55*	0.58***	0.78	0.92	0.60+	0.81	1.19	0.59*	0.77	0.62**	0.65*
Number of children <5 years	0.92	1.38*	0.91	1.15	0.84	0.95	0.80	0.91	0.89	0.88	1.04	0.99
Maternal education level:												
Never attended school	Ref	Ref	Ref	Ref	Ref	Ref	Ref	Ref	Ref	Ref	Ref	Ref
Primary school (Class 1–5)	1.23	1.55	1.49+	0.97	1.19	0.95	1.21	0.94	1.49+	1.02	1.42*	1.03
Middle school (Class 6–9)	1.39	2.05*	1.72*	0.98	1.33	1.05	1.32	1.57	1.63*	1.05	1.46*	1.17
High school or higher	1.28	2.35*	1.85*	0.77	1.66+	1.09	1.04	3.04	1.64*	0.99	1.65*	1.43
Occupation as housewife	1.19	1.00	1.02	1.42	1.36+	1.11	1.28	1.25	1.17	0.74+	1.28	1.11
TV ownership	1.14	1.22	1.05	2.16***	1.30**	1.50+	1.24	1.03	1.20	1.16	1.13	1.28*
Frequency of TV watching:												
Never	Ref	Ref	Ref	Ref	Ref	Ref	Ref	Ref	Ref	Ref	Ref	Ref
Infrequent (<3 days/week)	10.70***	1.01	8.44***	0.82	3.16***	0.78	13.17***	1.29	5.86***	0.62*	5.48***	1.24
Frequent (3+ days/week)	17.27***	1.60	15.11***	0.88	4.41***	1.17	19.67***	2.13+	9.94***	0.69+	9.75***	1.43
Received FHW visit	2.50***	1.82***	2.58***	2.01+	2.66***	1.49*	2.69***	1.71	2.41***	1.00	2.68***	1.57*

a Values are odds ratios [OR], adjusted for clustering at upazila level.

b Comprehension defined as identifying at least one correct message of the TV spot, among those who viewed the TV spot.

* *p* < .05.

** *p* < .01.

*** *p* < .001.

Among the factors associated with comprehension for multiple TV spots, older mothers also had significantly lower odds of comprehension (OR: 0.6–0.7; Table 4). Despite a pattern of increasing odds with higher education, education level was only significantly associated with comprehension of Spot 1 (OR: 2.4). TV ownership was associated with higher odds of comprehension for Spots 2 and 6 only (OR: 1.3 and 2.2, respectively). Mothers who received home visits from a FHW also had higher odds of comprehension (OR: 1.5–1.8).

### 3.3 Context of watching TV

As with the survey results, more than half of the respondents in the qualitative study reported watching TV (54%). However, TV watching was constrained by various factors within households, particularly time constraint due to work or chores (85%) and lack of access to TV or electricity (42%). Respondents cited a range of responsibilities that they needed to prioritize above TV viewing, including “outside work” for men and cooking and other household chores for women: “You know I have a little child. I have no time. Men come from the market to take their meals, so I have to cook. After doing all this, time is gone.” (mother, Sonaimuri). Men mentioned that they watched TV occasionally in the market or a tea stall, but many women were not able to watch TV in such extradomestic contexts.

Although many mothers explained that they can choose what they watch, some have been told not to watch TV by their parents or husbands, usually for religious reasons. TV watching was considered a “sin” or some types of programs were considered sinful or inappropriate. One mother mentioned that her father‐in‐law does not allow her to go outside the house to watch TV. In households where there are several members who want to watch different programs, not everyone can watch what they want. Some mothers reported limited decision‐making power about TV viewing, either because other family members restrict access to certain programs or they conform to watching whatever is on. In all three upazilas, films, dramas, and the news were mentioned as popular programs; respondents also referred to religious programming and, among men, sports.

During programming breaks or advertising pauses (when the TV spots are shown), many respondents recalled watching spots about child feeding, whereas others stated that they did not like to watch advertisements, preferred to shut the TV off during breaks to recharge the battery, or did not tend to give attention to advertising breaks because that time was used to carry out other tasks or necessary chores. Fathers were less likely to continue watching during programming breaks, with mothers showing greater receptiveness.

### 3.4 Message recalled, believability, identification, and feasibility

Responses to the messages recalled, believability, identification, and feasibility of the six TV spots are summarized in Table 5. In *Early Initiation of Breastfeeding* (Spot 1) and *Perception of Insufficient Milk* (Spot 2), there was strong believability, identification, and feasibility associated with the central messages, and many mothers expressed their ability to resist negative advice and pressures (*n* = 64), a behaviour that is modelled in these spots. Prior knowledge of these messages focused on breastfeeding was good; respondents from all upazilas noted that they had heard proearly initiation of breastfeeding, colostrum, and anti‐prelacteal messages from doctors, health workers, radio and TV, neighbours, friends, relatives, and other sources (*n* = 222). Mothers observed that health workers reinforced the same messages as those in the TV spots. However, advice coming from older women, particularly grandmothers/mothers‐in‐law, about feeding prelacteals such as honey, and the pressure to feed cow milk or formula/tinned milk from fathers (who were influenced by advertisements) and even doctors persisted as challenges. Furthermore, despite wide acceptance of breast milk being the best and sufficient for babies (*n* = 221), some mothers still struggled with perceptions and sensations of insufficient breast milk production. It is worth noting here that both Spots 1 and 2 used a direct, literal approach to narrative, in which the messages were clear and required no significant interpretive action or comprehension of visual metaphor from the viewer.

**Table 5:** Summary of qualitative results on comprehension, believability, identification, and feasibility of TV spots

TV spot	Comprehension (messages and elements recalled)	Believability, identification, and feasibility
1.Early initiation of breastfeeding (BF)	• BF was initiated within 1 hr of birth. • The mother depicted is smart because she asks for her baby immediately, so she can start BF. • Honey was suggested as a prelacteal but rejected by the mother in favour of BF. • Honey should not be administered as a prelacteal. • Mother hurries to give colostrum. • The importance of the medical authority figure in the TV spot.	Many mothers identified with the central messages, noted that it was credible and felt it was feasible to implement in their situations. Respondents in all upazilas reported familiarity with the message, from other sources. Examples of feasibility and challenges of behaviour: • One Chirirbandar mother first gave honey water to her youngest child because it took a few days for breast milk to fill her breasts. Later, she prevented others from feeding her child honey, and she continued to feed breast milk only. • One Sonaimuri mother did not give honey when her youngest child was born because she had heard from the TV spot and learned about correct BF practices. She also explained the practice to her family members so that prelacteals would not be given to the child.
2. Perception of insufficient milk	• The father is turned away when offering formula milk because breast milk is enough for the first 6 months. • Breast milk will make the child healthy. • Even a malnourished or sick mother can produce sufficient breast milk. • It was not well understood that the father should help with housework/chores so that the mother has time to breastfeed. Some viewers interpreted this as the mother needing to make time for BF.	This spot enjoyed wide credibility across the upazilas, where many people were already familiar with exclusive BF messages from other sources. Mothers identified with the dilemma confronted in the spot, and both mothers and fathers accurately interpreted the key messages communicated. Examples of feasibility and challenges of behaviour: • One Madhabpur mother was advised by her father‐in‐law to give *misri* water when the baby cried, but she refused all such requests and continued to breastfeed her child. • Most mothers were confident that they would be able to sufficiently breastfeed their child for 6 months. They were also prepared to oppose their husbands or anyone else suggesting tinned milk, by explaining the correct message.
3. Father's involvement	• “The child consumed nutritious foods and breastmilk (from early life). His brain was active. For that, he was able to advise his father during the fire.” (father, Chirirbandar) • Fathers need to buy nutritious food from the market for the mother to cook and feed the child. Then, the child will be healthy, strong, and intelligent. • Some respondents did not understand that quick thinking was a result of good nutrition; one Sonaimuri mother said, “nothing has entered into my brain; I haven't understood anything.”	The main message of the spot was not always understood. Where it was grasped, it was deemed credible and believable. Some viewers were able to explain the connection between fathers' involvement in providing nutritious foods and the child's good cognitive development. Examples of feasibility and challenges of behaviour: • Fathers who were unemployed or otherwise resource‐constrained explained that they were unable to consistently purchase nutritious food. • Many mothers reported having received practical support from their husbands in obtaining nutritious foods for children; this usually took the form of husbands purchasing such foods at the market.
4. Animal food	• The importance of feeding nutritious foods (viewers did not always mention animal‐source foods or list them). • Feed meat and fish. • The mother is feeding fish to the child. The doctor suggested feeding fish, eggs, and liver, so the baby will grow.	Viewers across the upazilas found it possible to identify with the characters and, in general, felt that the spot was credible. But opinions varied about the correct age to introduce animal source foods. Economic constraints were also expressed: Eggs and fish may be given more frequently, but meat is too expensive. Examples of feasibility and challenges of behaviour: • Most Chirirbandar parents said that egg or liver was easy to feed to the child as they are soft and do not have any bones. One mother noted that eggs are affordable and can be fed to the child nearly every day. • In Madhabpur, mothers also understood the message that feeding nutritious food such as fish, meat, and eggs is good for the child's well‐being, but few mothers did not think it was economically feasible to feed this every day.
5. Feeding quantity	• “… the child was fed nicely from childhood. And for that, she will do something good in the future.” (father, Chirirbandar) • Increase portions/amounts as the child gets older. • Feed nutritious foods such as leafy vegetables, and the child will be smart and good at sports. • “Yes, I watched this. Until 7 months, 2 times with half a bowl. Then after 9 months, 3 times with a half bowl. Then at 12 months, a full bowl for 3 times.” (mother, Sonaimuri)	This TV spot was challenging for respondents to understand and accept, likely in part because of the indirect messaging strategy and the different numerical information on offer all at once—how many bowls of food, how many times per day, at each age period. Examples of feasibility and challenges of behaviour: • In Chirirbandar, a majority of mothers and fathers did not feel that it was possible to feed the recommended amounts and frequencies. • In Madhabpur, respondents were confused about the numbers of food quantity, frequency, and age. • Some Sonaimuri respondents agreed that the recommendations are feasible, but many believed that three full bowls of food at 12 months was too much food.
6. Poor appetite	• “[I have to] bring fruits, vegetables, and good foods. Then, these foods should be fed to the child.” (father, Chirirbandar) • Do not fill the child's stomach with food from shops. Juice and cookies are bad for children. • Doctor tells parents not to fill the child's stomach with chips. A child's stomach is small. • Do not feed forcefully. The child will not eat by pressuring but will eat when hungry.	Poor appetite is a problem that many parents were able to identify with, but few made the connection between feeding junk food and poor appetite for meals. For practical reasons (i.e., ease and speed), some respondents fed their children unhealthy snacks or put pressure on them to eat. Examples of feasibility and challenges of behaviour: • In Chirirbandar, there was some disagreement about the need to pressure children to eat during meals. Lack of money and time were identified as barriers to preparing healthy snacks. • Forcing children to eat was also common in Madhabpur, and many parents continued to buy store snacks for their children. • Most respondents in Sonaimuri believed in and agreed with the message, but some continued to feed store‐bought snacks even if they know they should not. When a child is crying, it is easier to pacify quickly with a biscuit than to prepare a meal.

Similarly, the communication strategy of *Animal Food* (Spot 4) was direct and literal in its emphasis of the value of feeding animal source foods. Comprehension and believability were found to be good, although doubts about feasibility were expressed, either due to economic constraint (*n* = 63) or concerns about choking on fish bones or tough meats (*n* = 42). These results corroborate those of the survey results, which showed high comprehension of Spots 1, 2, and 4.

*Poor Appetite* (Spot 6) also employed a direct and literal narrative approach, and most respondents understood parts of the central message. However, the messages about not feeding junk foods and not force‐feeding were not fully understood by respondents. There was some confusion about what junk foods or store‐bought snacks are (or what is a nutritious snack) (*n* = 45) and their connection to appetite loss for cooked meals (*n* = 40), as well as the interpretation of feeding on demand (*n* = 30).

Spots 3 and 5 employed a storytelling approach or a more metaphorical and indirect narrative style to communicate their messages. Although the spots were designed to be culturally relevant and piloted for acceptability, this format was significantly less successful. Respondents had a difficult time identifying the main messages in *Father's Involvement* (Spot 3), which showed a child saving his father from a house fire. They struggled to make the connection between the house fire and better cognitive development from good nutrition (*n* = 85). Fathers are usually responsible for food purchasing, not only for the children but the entire family, but fathers' involvement in child feeding was not specifically pointed out as a spot message. Also, there was poor knowledge among fathers about what is nutritious food for children.

As with *Father's Involvement*, *Feeding Quantity*'s (Spot 5) indirect messaging about the frequency and quantity of feeding, conveyed through a storytelling scenario about a little girl who wins academic and sport prizes, made it difficult for respondents to recall and interpret the central messages (*n* = 65). *Feeding Quantity* was also made challenging by the relative density of numerical information provided. There were mixed responses about the actual recommendations as well as their believability and feasibility, as the feeding frequency and quantities of food were considered to exceed their realities (*n* = 32). Again, these findings corroborate the lower proportions of surveyed mothers who understood the messages for Spots 3 and 5.

## 4 DISCUSSION

Exposure to the TV spots in the intensive areas was moderate and consistently higher than in nonintensive areas. The factors associated with higher recall in viewing the TV spots were as expected—younger age, higher education, frequent TV watching, and receipt of home visits from a FHW (which was substantially higher in A&T‐intensive area, where media dark strategies were also implemented in specific hard‐to‐reach communities to increase exposure). However, despite more mothers in intensive areas reporting prior knowledge of the TV spot messages, there was no difference in comprehension between mothers in the program groups for most spots. Furthermore, the factors associated with viewing were not necessarily associated with comprehension. Younger age and receipt of home visit from FHWs were associated with higher comprehension for multiple spots, but higher education and frequent TV watching were not. TV ownership, which likely influences what is watched in addition to how often, was associated with higher comprehension for two spots.

Spots 1, 2, and 4 (*Early initiation of breastfeeding*, *Perception of insufficient milk*, and *Animal food*, respectively) had the highest rates of exposure and comprehension, whereas Spots 3, 5, and 6 (*Father's involvement*, *Feeding quantity*, and *Poor appetite* respectively) had the lowest rates of exposure, prior knowledge, and comprehension. A review of the reports of monthly airing from the mass media firms showed that Spot 3 was aired significantly less compared with the other five spots, particularly after the first year, but the other five spots were aired with similar frequencies. The qualitative findings revealed strong believability, identification, and feasibility for Spots 1, 2, and 4. Although Spot 6 employed a direct and literal narrative approach, the concepts about not feeding junk foods and not force‐feeding were not fully understood by respondents. Spots 3 and 5 employed a more metaphorical and indirect narrative style to communicate their messages, and the central messages appeared difficult for respondents to identify. Spot 5 also contained dense numerical information about feeding quantity, which respondents had difficulty in remembering correctly, and some believed that the recommended frequency and quantity were not feasible.

Direct narrative messaging appeared to lead to better comprehension than indirect narrative forms in our context, and messaging was most effective when it is combined with complementary activities such as home visits by FHWs to deliver similar messages. Although metaphors are commonly used in persuasive message design, our findings concur with those from other mass media interventions, suggesting simple well‐developed messages for target audiences (Snyder, 2007) and implementation with combined behaviour change strategies contribute to successful interventions (Nguyen et al., 2016; Sinha et al., 2015).

Even with high believability and identification with the situation or characters, the barriers to feasibility such as sociocultural issues and economic constraints continue to present obstacles to implementing behaviours and may undermine the uptake of messages into practice. Thus, for behaviours highly embedded in sociocultural contexts such as child feeding practices, mass media interventions that reach caregivers as well as other family members and the broader community play an important role in combination with other strategies in promoting IYCF practices (Menon et al., 2016; P. H. Nguyen, Kim, et al., 2016), and more evidence such as our study is needed in support of refining these interventions for social and behaviour change. In our study, we examined the various factors (Salmon & Atkin, 2003), with the assumption that these elements work together to influence behaviour. Thus, the lack of any one element—message comprehension, believability, identification, or feasibility—may hinder behaviour change.

There are limitations to this study. During the interview process, respondents were shown the six TV spots in series, so there may have been some knowledge accumulation during the interview process (i.e., information from one TV spot may have given suggestions for or triggered responses about subsequent TV spots shown). However, we did not observe patterns of increasing rates of spot recall and comprehension (i.e., higher recall or correct responses in later spots), and the interviewers were trained and skilled at survey and qualitative research techniques, in order minimize these biases. Although we found that indirect narrative styles and dense numerical information led to poorer recall and comprehension of messages, we do not know whether these improve with repeated viewing. Nonetheless, we found many of our findings to be salient even among those who had seen the TV spots before. Lastly, it is possible that comprehension, as measured by recall of the main messages, is a result of rote learning (or memorization) rather than meaningful learning. Although we did not examine whether message comprehension was due to rote or meaningful learning, we acknowledge that comprehension does not act alone to influence behaviour, thus understanding the correct messages in either case needs to be supported by other factors that relate to further internalizing the messages in order to lead to behaviour change.

The most significant implication for mass media interventions is that direct narrative messaging works best for comprehension in our context. This comes across unequivocally in both the quantitative survey and qualitative work. Messaging also appears to be more effective when it is synergistic—working in harmony with similar messages particularly from contacts with FHWs. Finally, we note that the sociocultural context of TV watching (e.g., religious beliefs, decision‐making power over TV viewing, and viewing habits) limits exposure to mass media interventions and issues of feasibility, together with economic constraints, continue to present obstacles to adoption of even well‐promoted and well‐understood IYCF practices. Understanding the differences in the uptake factors may help to explain variability of impacts and ways to improve the design and implementation of mass media strategies.

## CONFLICTS OF INTEREST

The authors declare that they have no conflicts of interest.

## CONTRIBUTIONS

SSK, TR, KKS, and PM contributed to the study design. SSK, TR, MIB, and PHN conducted the data analysis. SSK and TR drafted the manuscript. SSK, TR, PHN, and PM critically reviewed and edited the manuscript. All authors read and approved the final version of the manuscript.
